# The mitochondrial genome sequence of a satyrid butterfly, *Lethe confuse* (Lepidoptera: Nymphalidae: Satyrinae)

**DOI:** 10.1080/23802359.2020.1791017

**Published:** 2020-07-15

**Authors:** Xiao-Dong Li, Wei Zhang, Wan-Tao Rong, Shuo-Feng Wang, Ran Li

**Affiliations:** a School of Chemistry and Bioengineering, Hechi University, Yizhou, Guangxi, P. R. China; b The Key Laboratory of Jiangsu Biodiversity and Biotechnology, College of Life Sciences, Nanjing Normal University, Nanjing, Jiangsu, P. R. China

**Keywords:** Lepidoptera, Nymphalidae, *Lethe confuse*, mitogenome, phylogenetic analysis

## Abstract

The nearly complete mitochondrial genome (mitogenome) of *Lethe confuse* was sequenced and analyzed. This mitogenome is 14,945 bp in size and encodes 13 protein-coding genes, 22 transfer RNA genes, 2 ribosomal RNA genes. The most common start codon is ATN (ATA, ATG, and ATT), and the most common termination codon is TAA. In addition, three PCGs have incomplete termination codon T. The overall nucleotide composition is 38.0% of A, 7.8% of G, 42.4% of T, and 11.8% of C. The data will increase the basic information of Satyrinae phylogenetic research and can help to better understand the phylogenetic status of *L. confuse* in Nymphalidae.

The subfamily Satyrinae, one of the diverse groups of Nymphalidae (12 subfamilies), is distributed worldwide with more than 2500 described species (Pena and Wahlberg 2008). Due to the limited molecular information, there have been no robust hypotheses on the phylogenetic relationships of Satyrinae . Recent researches showed mitogenome is an effective molecular marker in phylogeny analysis among Lepidoptera (Yang et al. 2019). In the current study, we sequenced the mitochondrial genome of *Lethe confuse* (GenBank accession No. MT654529), which will help to better understand the phylogenetic relationships among Satyrinae.

Adult specimens of *L. confuse* were collected in Luocheng in Guangxi Zhuang Autonomous Region, China. The collected specimens were then stored in 95% ethanol at temperature −20 °C. Whole genomic DNA was extracted from the right middle leg of an adult specimen using a Wizard^®^ Genomic DNA Purification Kit (Promega, Madison, WI) according to the manufacturer’s instructions. The specimens and DNA of these specimens were deposited in the Museum of Insects of Hechi University (the voucher No. L388), Yizhou, Guangxi. The genomic DNA was sequenced using the Hiseq2500 platform (Illumina, San Diego, CA). The mitogenome of *Lethe sicelis* (GenBank accession No. LC541741) was employed as the reference sequence (Nagata et al. 2020). The mitochondrial genome was assembled by Geneious 9.0.4 (https://www.geneious.com) and annotated using MITOS Web Server (Bernt et al. 2013).

We obtained the partial mitogenome of *L. confuse* with 14,945 bp long. The region that we failed to sequence was between *rrnS* and *trnM*, and generally contained a putative control region. This mitogenome encoded 13 protein-coding genes (PCGs), 22 tRNAs, and two ribosomal RNAs (*rrnL* and *rrnS*). The overall nucleotide composition was 38.0% of A, 7.8% of G, 42.4% of T, and 11.8% of C. Twelve PCGs started with typical ATN codon (two with ATA, three with ATT and seven with ATG), whereas the *cox1* gene appeared to start with CGA. Ten PCGs terminated with TAA, and the remaining three terminated with an incomplete stop codon T. The 22 tRNA genes ranged in size from 60 to 71 bp. The *rrnS* (772 bp) and *rrnL* (1361 bp), were located between the *trnL1* and AT-rich region, and separated by the *trnV* gene.

To validate the phylogenetic position of *L. confuse*, the BI and ML trees were constructed on CIPRES Portal using 13 mitochondrial PCGs from mitogenomes of 27 Satyrinae species and two outgroups of Danainae, respectively (Ronquist et al. 2012; Stamatakis 2014). We used the best-fit partitioning scheme and partition-specific models recommended by PartitionFinder (Lanfear et al. 2012). Two phylogenetic analyses using different methods yielded the same topology, and nodal supporting values were always higher for BI tree than for ML tree. As shown in Figure 1, *L. confuse* was positioned near *L. sicelis* within the genus of *Lethe*. The phylogenetic tree indicated that the genus *Lethe* was a monophyletic group in Satyrinae.

**Figure 1.**
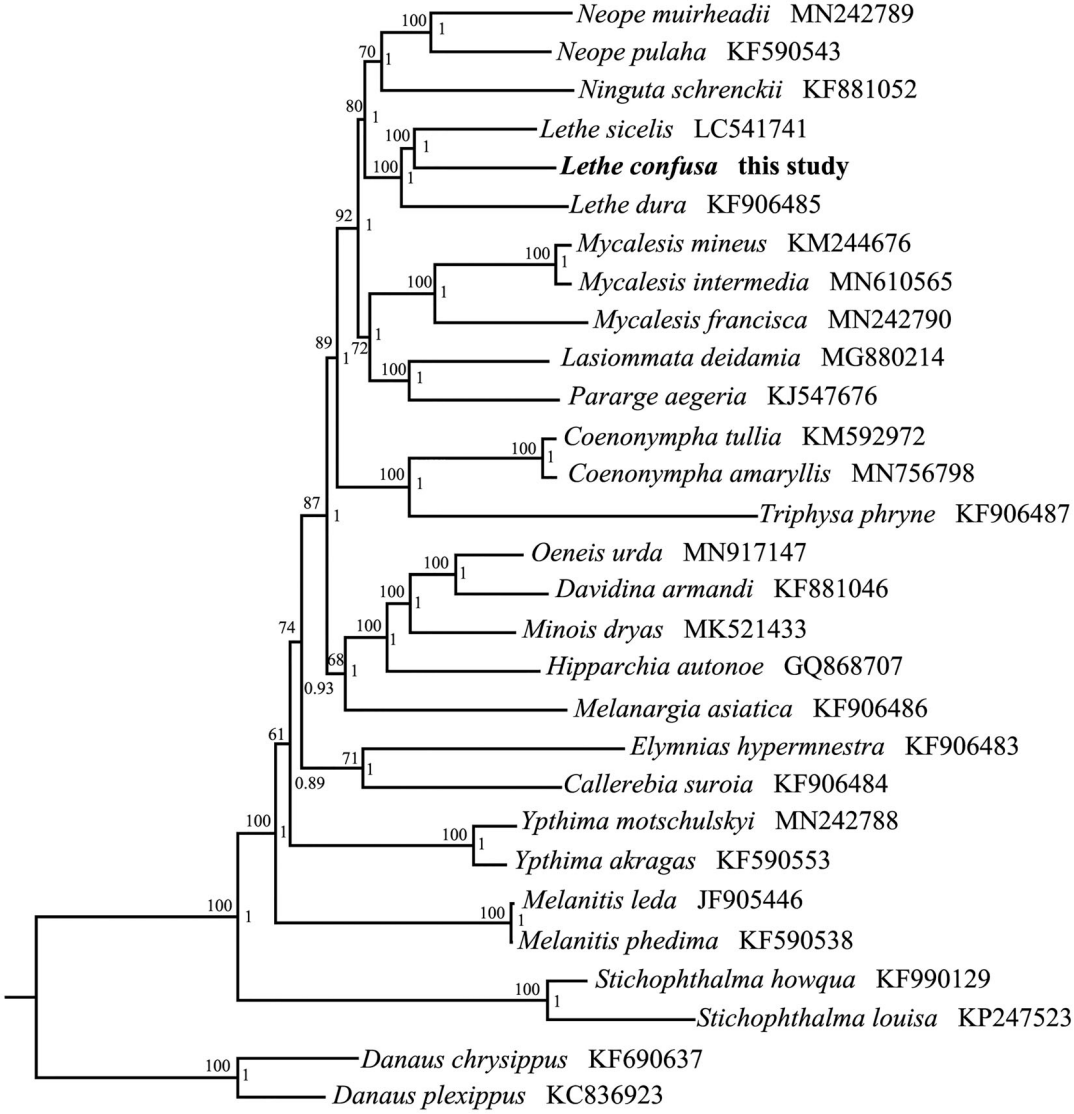
Phylogenetic tree obtained from ML and BI analysis based on 13 concatenated mitochondrial PCGs. Numbers on node are posterior probability (PP) and bootstrap value (BV).

## Data Availability

The data that support the findings of this study are openly available in National Center for Biotechnology Information at https://www.ncbi.nlm.nih.gov/nuccore, reference number (MT654529).
